# Supplementary material for: Cdt1 stabilizes an open MCM ring for helicase loading
Source: Nat Commun. 2017 Jun 23;8:15720. doi: 10.1038/ncomms15720 (PMC5490006; doi:10.1038/ncomms15720)
Supplement: Supplementary Information [file ncomms15720-s1.pdf]

Description of Supplementary Files

File name: Supplementary Information
Description: Supplementary figures, supplementary tables, supplementary methods and supplementary references.

File name: Supplementary Movie 1
Description: Building a MCM-Cdt1 molecular model. Docking of MCM and Cdt1 atomic structures into the ATPγS MCM-Cdt1 EM density.

File name: Supplementary Movie 2
Description: MCM loading promotes Cdt1 ejection. Structural transition from the pre-catalytic MCM-Cdt1 to the post-catalytic (loaded) MCM involves ORC-Cdc6 mediated MCM planarization and ATP-hydrolysis AAA+ tier rotation, resulting in closure of the Mcm2-5 gate and Cdt1 ejection.

File name: Peer review file

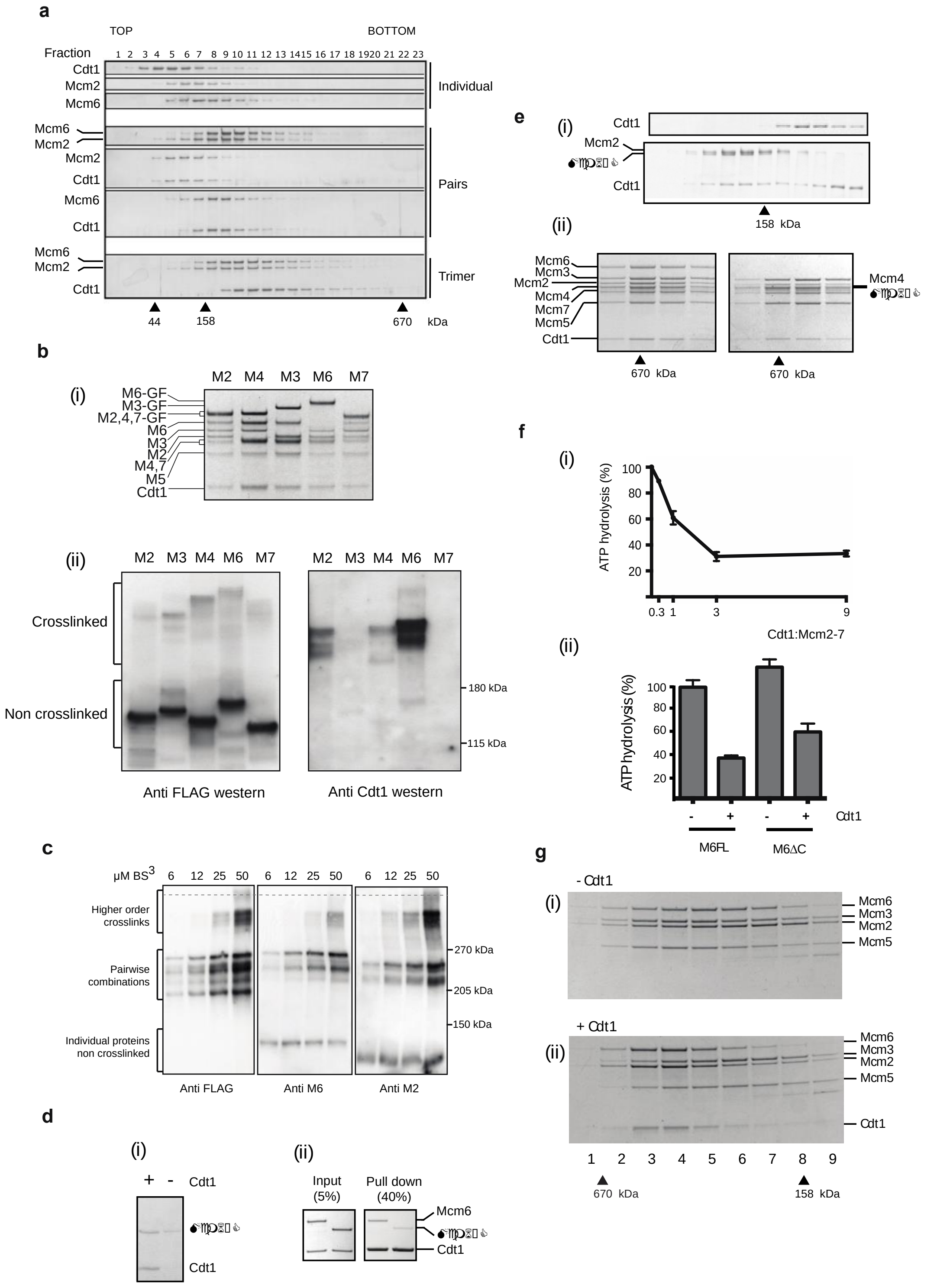
a
TOP
BOTTOM
Fraction
Cdt1
Mcm2
Mcm6
Individual
Pairs
Trimer
44
158
670
kDa
b
(i)
M2 M4 M3 M6 M7
M6-GF
M3-GF
M2,4,7-GF
M6
M3
M2
M4,7
M5
Cdt1
(ii)
M2 M3 M4 M6 M7
Crosslinked
Non crosslinked
180 kDa
115 kDa
Anti FLAG western
Anti Cdt1 western
c
μM BS3
6 12 25 50
Higher order crosslinks
Pairwise combinations
Individual proteins non crosslinked
270 kDa
205 kDa
150 kDa
Anti FLAG
Anti M6
Anti M2
d
(i)
Cdt1
(ii)
Input (5%)
Pull down (40%)
Mcm6
Cdt1
e
Mcm3
Mcm4
Mcm7
Mcm5
158 kDa
670 kDa
f
ATP hydrolysis (%)
Cdt1:Mcm2-7
0.3 1 3 9
M6FL
M6ΔC
g
- Cdt1
+ Cdt1
1 2 3 4 5 6 7 8 9
670 kDa
158 kDa

Supplementary Figure 1

(a) Cdt1 forms stable trimer with Mcm2 and 6. Glycerol Gradient sedimentation of individual Cdt1, Mcm2 and Mcm6 proteins (upper panels); Pairs of Mcm2-Mcm6, Mcm2-Cdt1 and Mcm6-Cdt1 (middle panels); Trimer of Mcm2-Mcm6-Cdt1 (bottom panel). (b) Crosslinking of Cdt1 with different MCM complexes, where individual Mcm have been double tagged with GFP and FLAG (MCM GFP,FLAG-Cdt1 complexes).M's above gels denote which Mcm has been tagged in the MCM-Cdt1 complex. (i) Silver staining of the different complexes (GF on the left indicates position where tagged individual Mcm's run on the gel). (ii) Immunoblot analysis of the crosslinked complexes. Anti-FLAG antibody used as an input control and anti-Cdt1 shows which MCM GFP,FLAG-Cdt1 was able to crosslink Cdt1 after anti FLAG denaturing IP. (c) Identification by immunoblotting of different Mcm-Cdt1 FLAG pairwise combinations showed in Figure 1b (anti FLAG denotes Cdt1 and anti M6 and M2 show crosslinked Mcm6 and 2 with Cdt1, respectively). (d) Cdt1 is able to interact with Mcm6ΔC. (i) FLAG pull down analysis of Cdt1 FLAG with M6ΔC. Minus Cdt1 sample was used as a negative control. (ii) Side by side pull down analysis of Cdt1 FLAG with Mcm6 fl and ΔC. (e) Cdt1 forms stable higher molecular complexes with Mcm6 ΔC (i). Gel filtration analysis of individual Cdt1 (upper panel) and together with Mcm2 and 6 ΔC (lower panel). . (ii) Cdt1 forms a higher molecular weight complex with both Mcm6 fl (left) or ΔC (right), as shown by gel filtration. (f) Cdt1 inhibits ATPase activity of MCM complex. (i) Inhibition of MCM ATPase activity with increasing molar ratios of Cdt1:MCM. (ii) Cdt1 inhibits activity of both MCM complexes (Mcm6 fl and ΔC). The assays were done at Cdt1:MCM molar ratio of 3. (g) Gel filtration analysis of stable tetrameric Mcm6-2-5-3 complexes, with or without Cdt1.

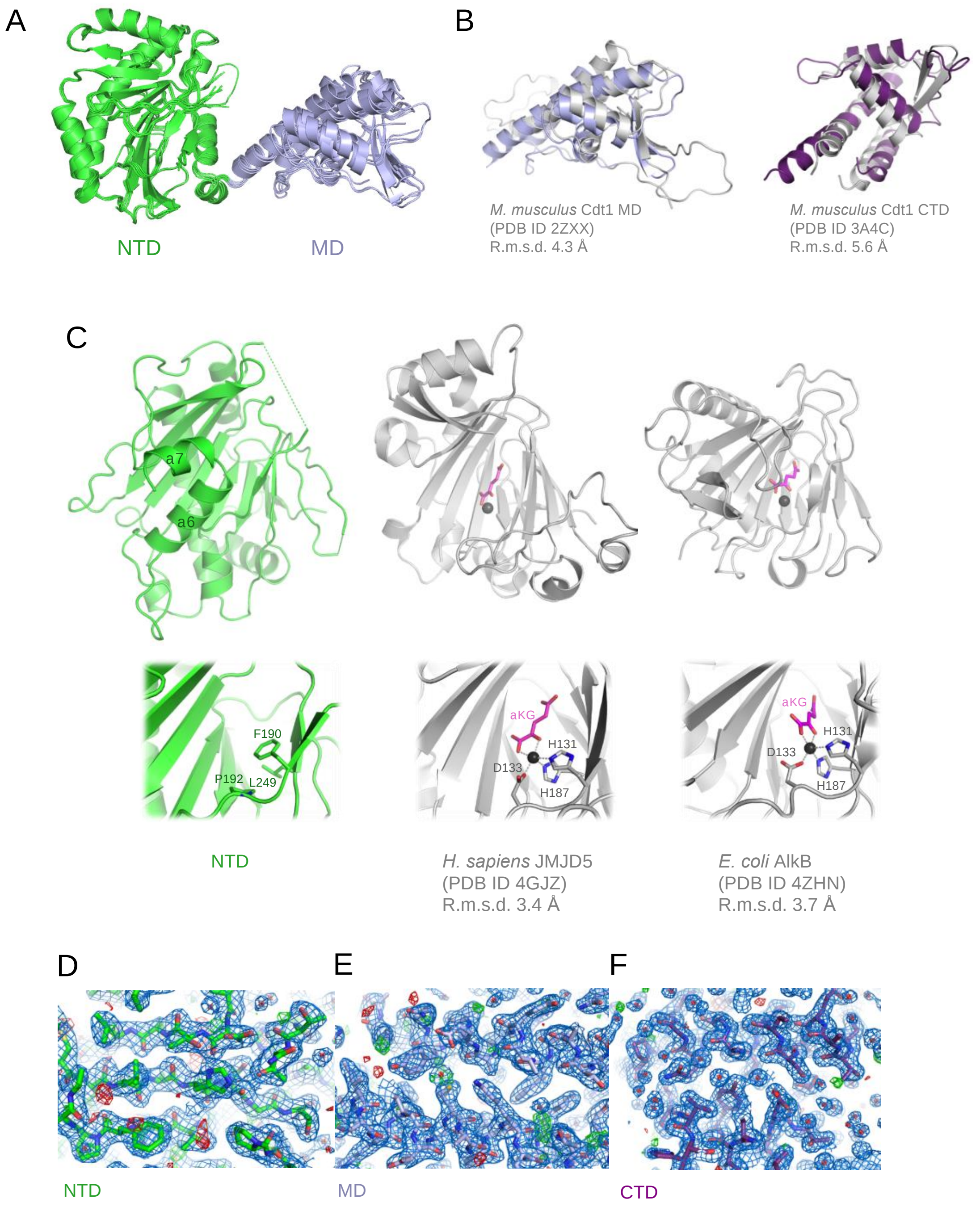

Supplementary Figure 2

Superposition of four independently observed protein chains in crystals of Cdt1(1-438, NM); root mean square deviation (r.m.s.d.) values for Cα atom positions in pairwise superpositions of the chains ranged between 0.46 and 1.1 Å. The domains are colored as in Figure 2. (B) Superposition of the *S. cerevisiae* with *M.*

musculus Cdt1 M domain (left) and CTD (right) WHD domains; the mouse Cdt1 structures are shown in grey. (C) Comparison of the *S. cerevisiae* Cdt1 N domain structure (left), human Lys demethylase JMJD5 (PDB entry 4GJZ, middle) and *E. coli* AlkB DNA dealkylase (PDB entry 4ZHN, right). Bottom panels show zoomed views corresponding to active site area of the dioxygenase fold, with selected residues in sticks and indicated. Grey spheres are metal ions (represented by Co^{2+} in the JMJD5 and AlkB structures), and dashes indicate coordination bonds; α-ketoglutarate is shown as sticks with carbon atoms in magenta. Note that in the Cdt1 N domain, the critical His and Asp residues involved in metal ion chelation (His131, Asp133, and His187 in JMJD5 and AlkB) are replaced with amino acid residues not capable of metal chelation (Phe190, Pro192 and Leu249, respectively). (D-F) Representative electron density maps of the Cdt1 crystal structures. 2Fo-Fc maps are contoured at 1 sigma (blue); Fo-Fc maps are contoured at 3 sigma (positive, green; negative, red). D. shows Ctd1 N-terminal in green sticks. E. shows Cdt1 middle domain in light blue sticks. F. shows Cdt1 C-terminal domain in violet sticks.

A

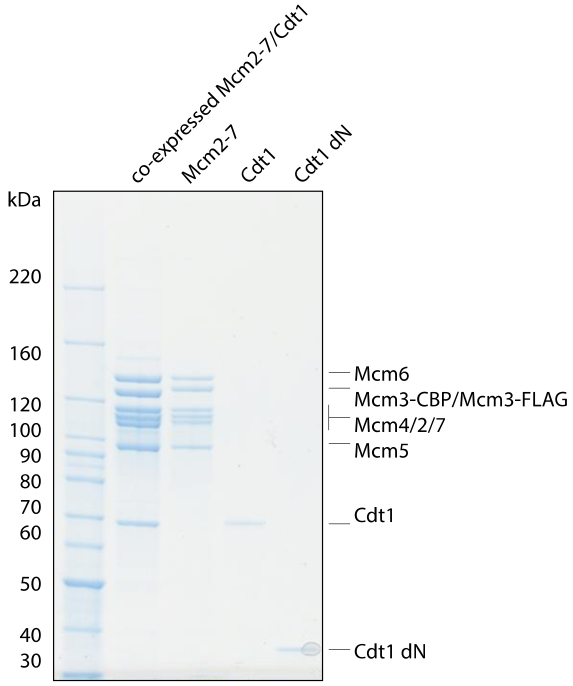
co-expressed Mcm2-7/Cdt1
Mcm2-7
Cdt1
Cdt1 dN
kDa
220
160
120
100
90
80
70
60
50
40
30
Mcm6
Mcm3-CBP/Mcm3-FLAG
Mcm4/2/7
Mcm5
Cdt1
Cdt1 dN

B

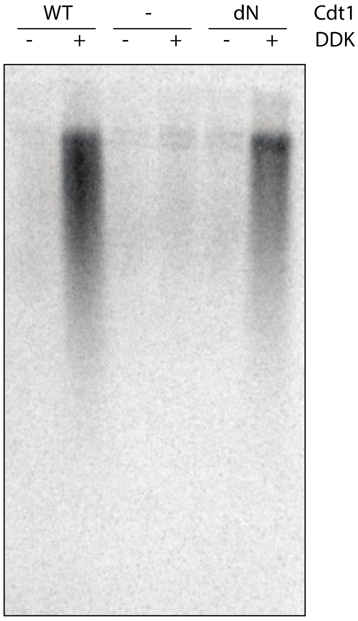
WT
-
dN
Cdt1
- + - + - +
DDK

Supplementary Figure 3.

(A) Coomassie-stained SDS-PAGE of purified proteins used to reconstitute Cdt1-MCM complexes: co-expressed Cdt1-MCM (lane1), Mcm2-7 (lane 2), Cdt1 WT (lane 3), Cdt1MC (lane 4).

(B) Alkaline agarose gel of DDK-dependent replication products after *in vitro* replication assays using S-phase whole-cell extract. Cdt1-MCM complexes were reconstituted with either WT Cdt1 (lanes 1+2), no Cdt1 (lanes 3+4) or Cdt1MC (lanes 5+6). Differences in loading efficiency were not corrected, resulting in reduced replication efficiency in the presence of Cdt1MC (lane 6), and complete loss of replication when omitting Cdt1 (lane 4), compared to WT Cdt1 (lane 2).

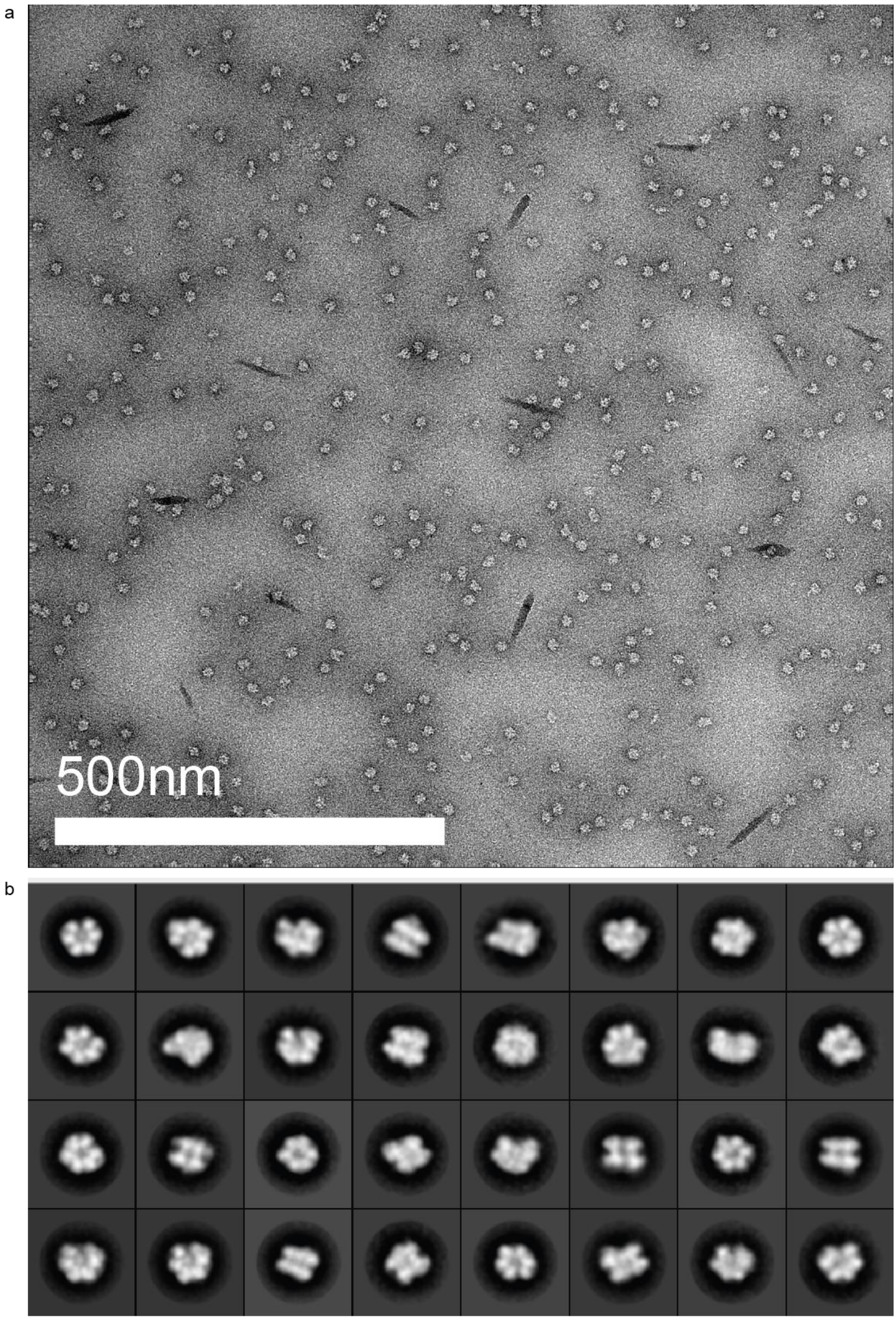
a
500nm
b

Supplementary Figure 4. Electron microscopy imaging of apo MCM particles.

(A) Characteristic micrograph of negatively stained MCM particles. (B) Two-dimensional classification reveals the presence of a mixed population of open and closed MCM rings.

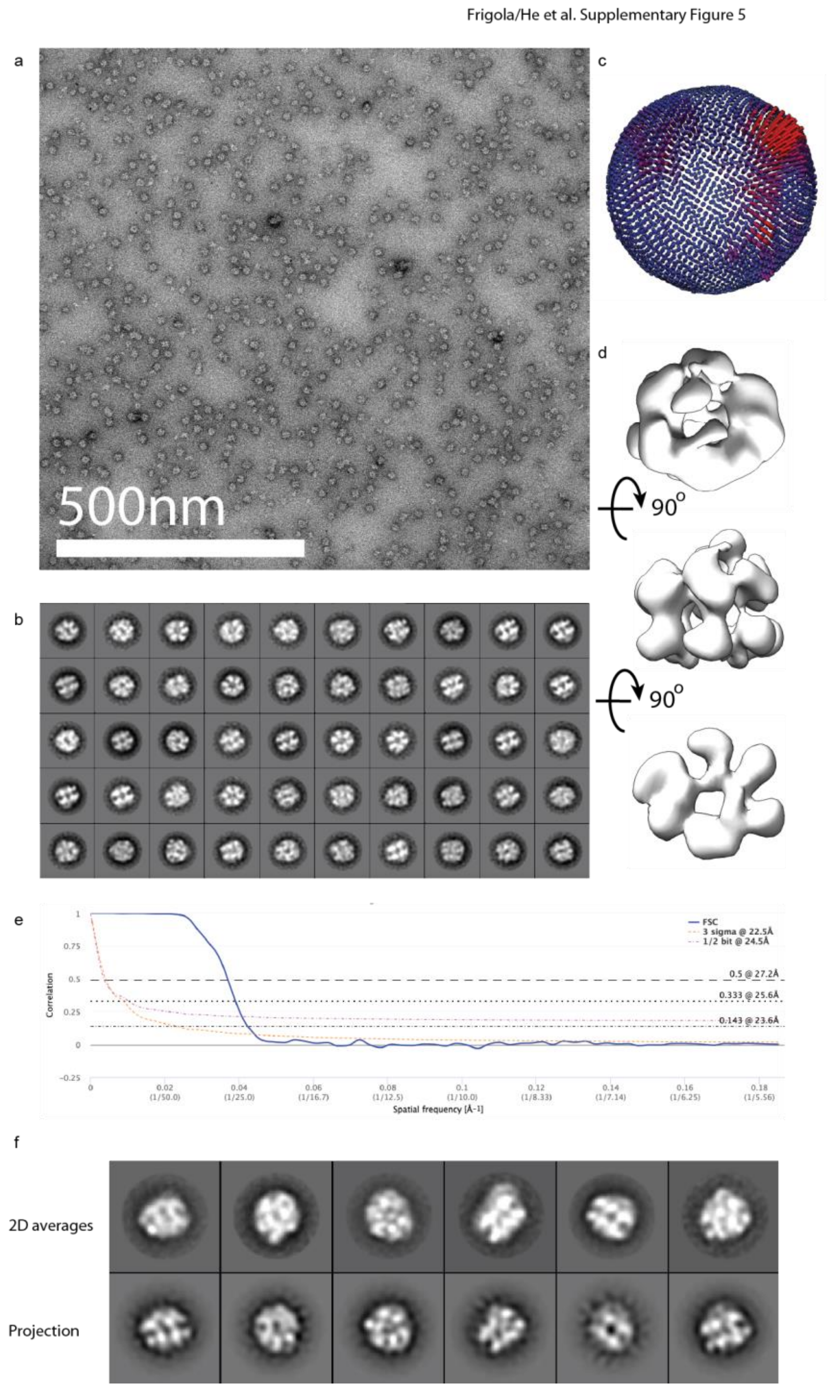

Supplementary Figure 5. Electron microscopy imaging of ATPγS-MCM particles.

(A) Characteristic micrograph of negatively stained MCM particles. (B) Two-dimensional classification indicates that ATPγS binding stabilises a closed configuration of MCM rings. (C) Euler angle distribution. (D) Three-dimensional

structure of ATPγS-MCM reveals a closed planar ring. (E) Fourier-shell correlation indicates a resolution of 23.6 Å according to the 0.143 criterion. (F) Match between reverence free class averages and 2D re-projections.

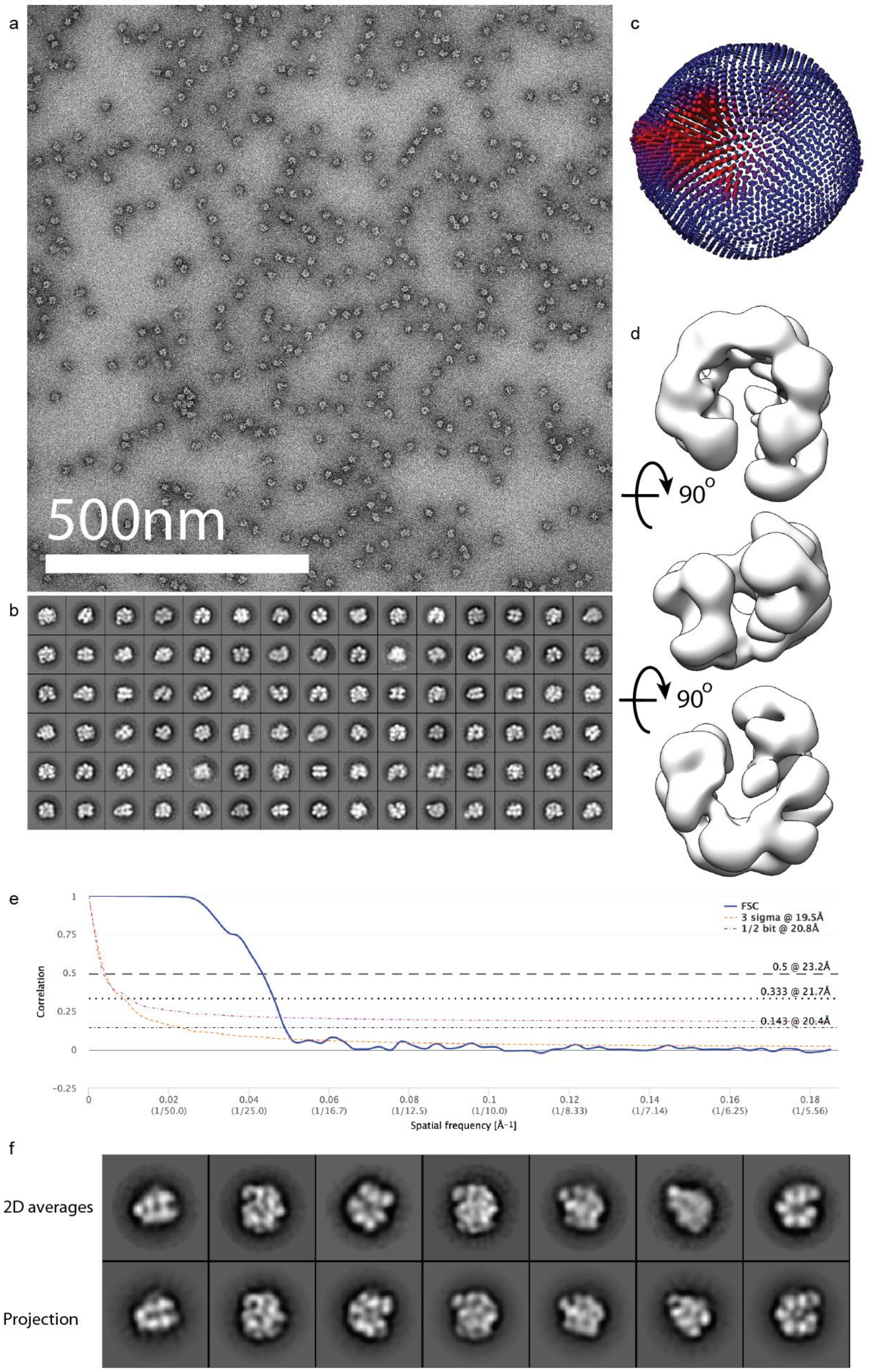
a
500nm
b
c
d
90°
90°
e
FSC
3 sigma @ 19.5Å
1/2 bit @ 20.8Å
0.5 @ 23.2Å
0.333 @ 21.7Å
0.143 @ 20.4Å
Correlation
Spatial frequency [Å-1]
f
2D averages
Projection

Supplementary Figure 6. Electron microscopy imaging of apo Cdt1-MCM.

(A) Characteristic micrograph of negatively stained particles. (B) Two-dimensional classification indicates that Cdt1-bound, apo MCM particles form stable open rings. (C) Euler angle distribution. (D) Three-dimensional structure of apo Cdt1-MCM reveals a left-handed spiral MCM structure. (E) Fourier-shell correlation indicates a resolution of 20.4 Å according to the 0.143 criterion. (F) Match between reverence free class averages and 2D re-projections.

Frigola/He et al. Supplementary Figure 7

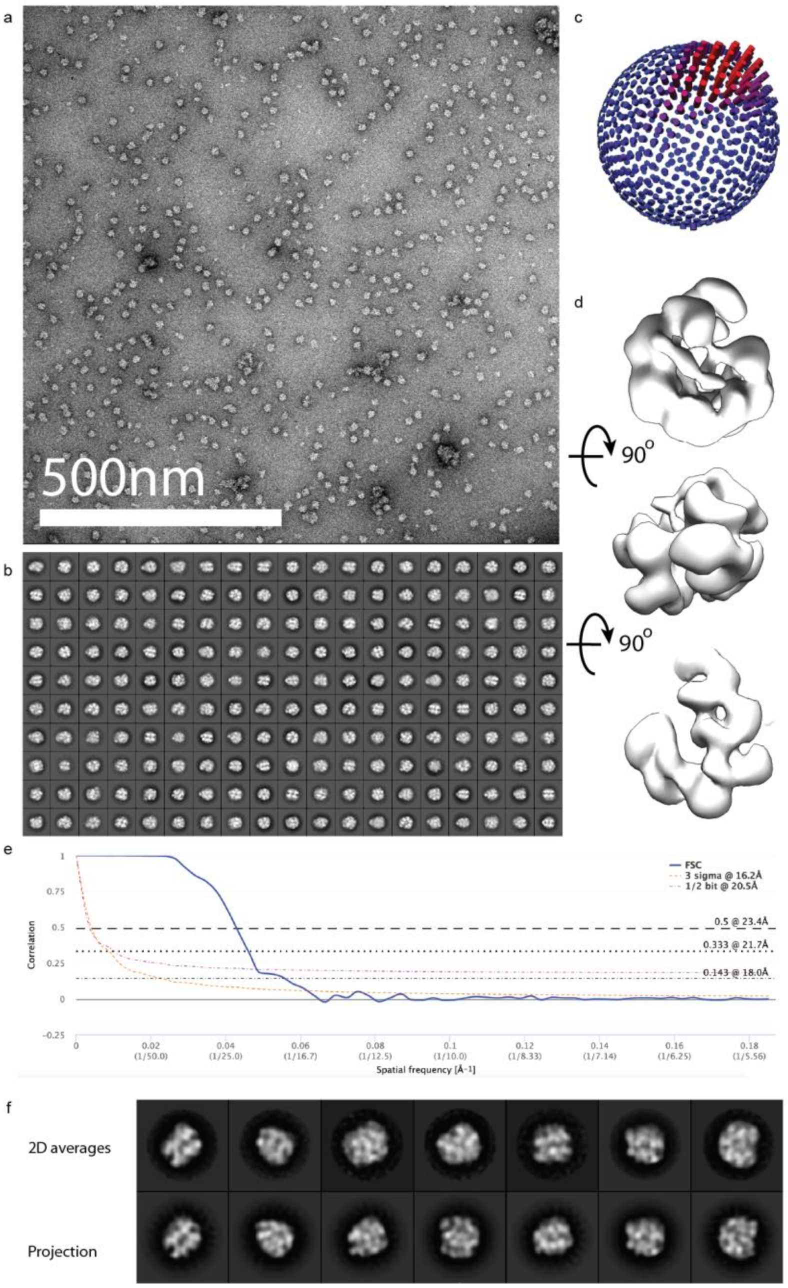

Supplementary Figure 7. Electron microscopy imaging of ATPγS-Cdt1-MCM.

(A) Characteristic micrograph of negatively stained particles. (B) Two-dimensional classification indicates that Cdt1-bound, ATPγS-MCM particles form stable open rings. (C) Euler angle distribution. (D) Three-dimensional structure of ATPγS-Cdt1-MCM reveals a left-handed spiral MCM structure. (E) Fourier-shell

correlation indicates a resolution of 20.4 Å according to the 0.143 criterion. (F) Match between reverence free class averages and 2D re-projections.

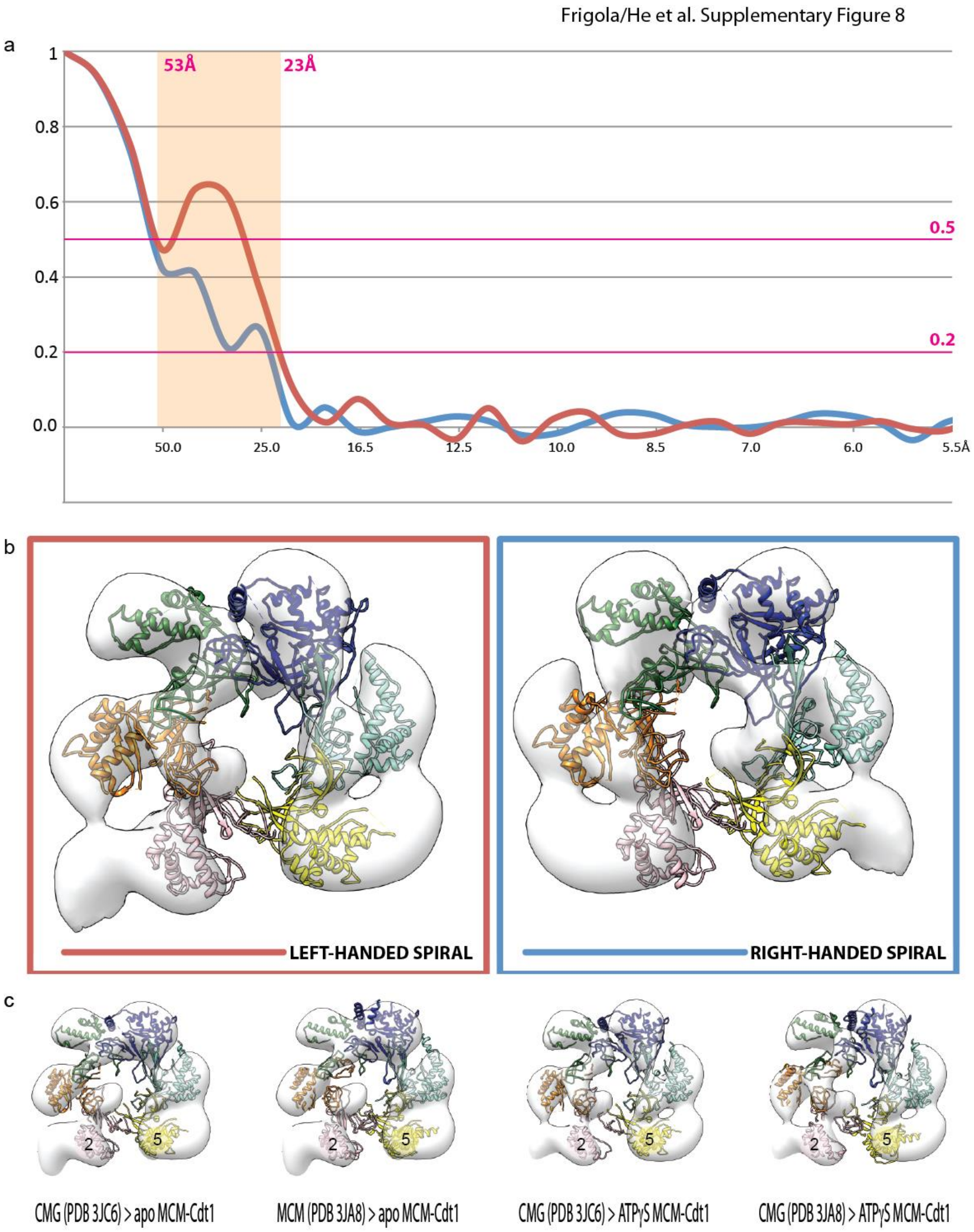

Supplementary Figure 8: handedness and ring subunit register of the ATPγS-Cdt1-MCM structure.

(A) Fourier-shell correlation between the N-terminal domain of MCM and the atomic structure of the yeast MCM N-terminal domain (extracted from the CMG

atomic model, PDB entry 3JC6). A left handed MCM spiral has higher correlation compared to a right-handed spiral, in the 53-23 Å resolution range (0.5-0.2 correlation interval). (B) The best docking solutions of the two mirrored N domain-MCM EM maps. (C) UCSF Chimera automated fitting of the atomic model of N domain-MCM extracted from the CMG or the MCM double hexamer, into the apo- or ATPγS-bound Cdt1-MCM indicates the same MCM ring subunit register (MCM gate maps between Mcm5 and Mcm2).

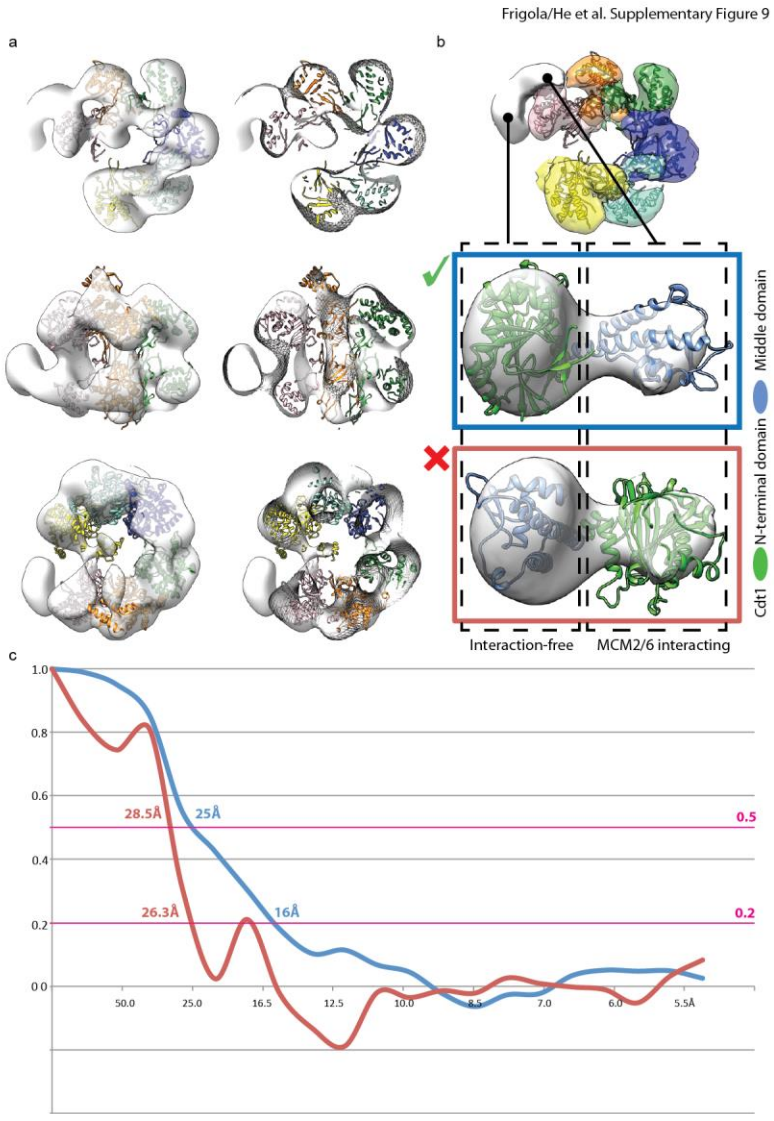

Supplementary Figure 9. Cdt1-MCM atomic docking.

(A) Local adjustment of MCM domains performed to accurately model the open ring configuration. Mcm2-6-4 N domain, Mcm2-6-4 AAA+, Mcm7-3-5 N domain

and Mcm7-3-5 AAA+ were treated as four independent rigid bodies. (B) Unoccupied density was assigned to Cdt1 and can be used for docking the structure of the N domain/middle domain of Cdt1. The best docking solution indicates that the N domain is free of any MCM interactions, while the middle domain interacts with Mcm2-6 (compatible with the results of the domain mapping experiment reported in Figure 1e). An alternative docking, with a lower-score, and Cdt1 rotated ~180°, shows the N domain-Cdt1 contacting the MCM and the middle domain free of any MCM interaction. This second solution is incompatible with the domain mapping experiments. (C) Fourier shell correlation of the Cdt1 EM density vs an artificial electron density map generated from the docked coordinates. The best solution consistently gives a higher correlation across a large resolution range.

Frigola, He et al. Supplementary Figure 10

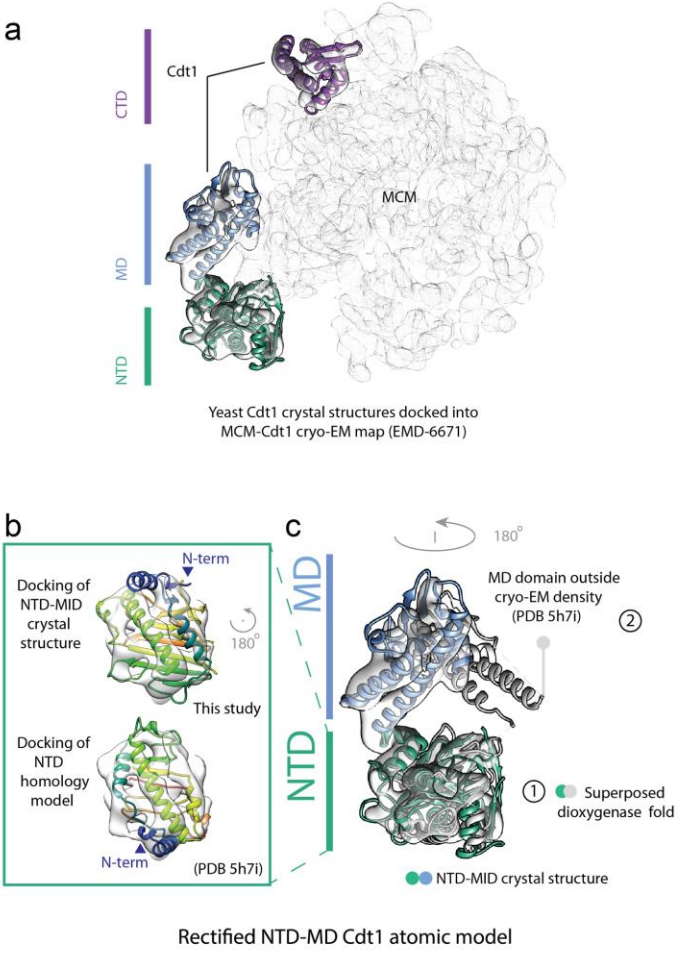

Supplementary Figure 10. Comparison between the Cdt1 atomic models based on crystallography and cryo-EM. The NTD/MD and the CTD crystallographic structures of yeast Cdt1 can be docked into the ~7.1 Å resolution cryo-EM structure of yeast AMPPNP-MCM-Cdt1 (EMD-6671). The best docking solution for NTD/MD Cdt1 agrees with that based on our negative stain ATPγS-MCM-Cdt1. The crystallographic and cryo-EM atomic models of NTD-Cdt1 disagree, in that the dioxygenase fold is ~180° rotated. As a consequence, the connectivity between NTD-MD Cdt1 is different and superposition of the two atomic model aids in clarifying this discrepancy. Docking of the NTD/MD Cdt1 crystal structure into the cryo-EM map is unequivocal, however NTD-based superposition of the previously published NTD-MD Cdt1 atomic model (PDB entry 5h7i) to the docked crystal structure shows that the MD-Cdt1 resides outside of the cryo-EM density.

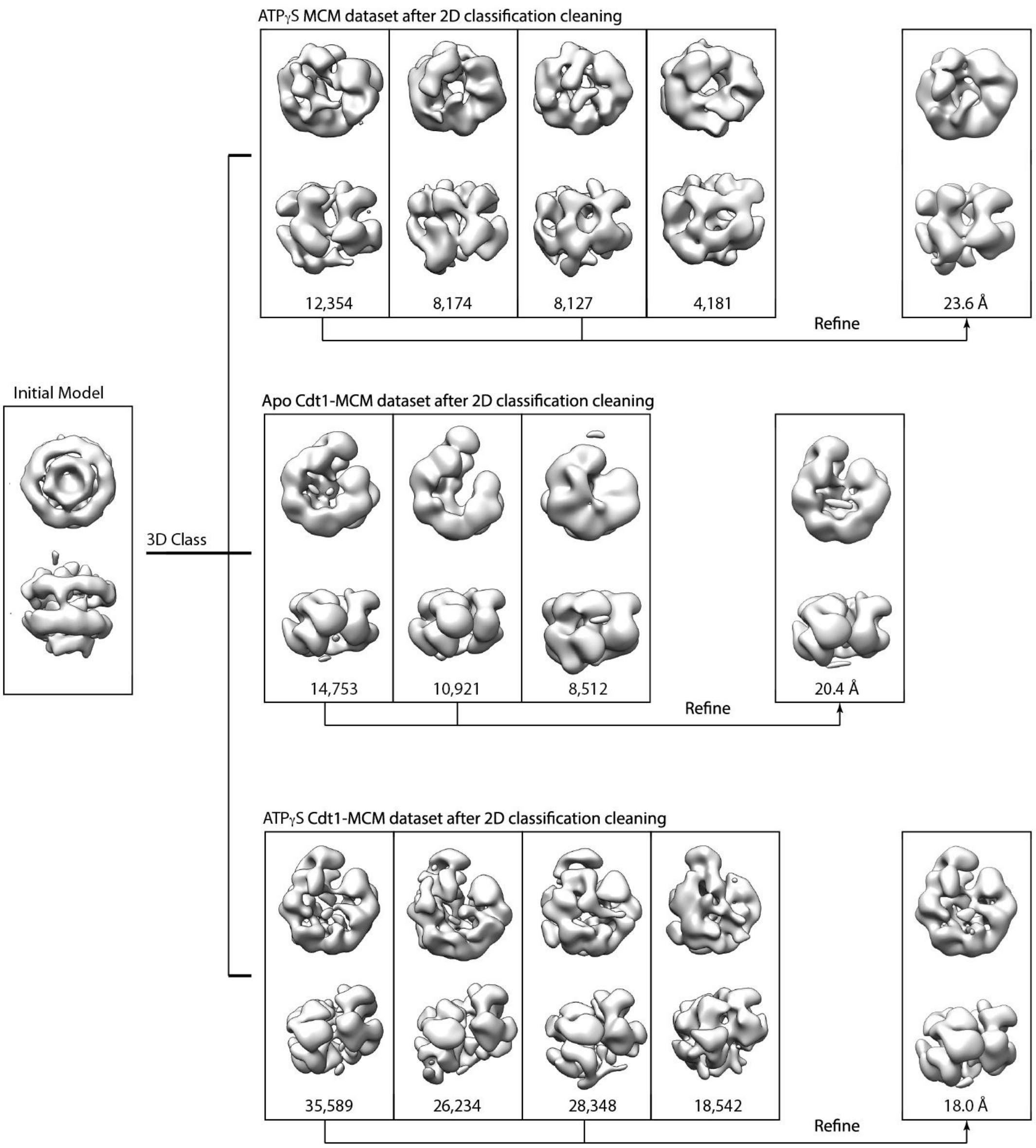
ATPγS MCM dataset after 2D classification cleaning
12,354
8,174
8,127
4,181
23.6 Å
Refine
Initial Model
3D Class
Apo Cdt1-MCM dataset after 2D classification cleaning
14,753
10,921
8,512
20.4 Å
Refine
ATPγS Cdt1-MCM dataset after 2D classification cleaning
35,589
26,234
28,348
18,542
18.0 Å
Refine

Supplementary Figure 11. Overview of the three-dimensional classification and structure refinement. The initial model is a planar MCM ring (i.e. the asymmetric unit from the negative stain structure of the MCM double hexamer[1]). Particles contributing to the two better-defined 3D classes in each dataset were used for 3D refinement.

Frigola/He Supplementary Table 1

#	Visible?	Starred?	Identified Pr	Accession Nu	Molecular We	Protein Grou	Quantitative	Taxonomy	Uncategorize BioSample 1 CSM4589A1F	Uncategorize BioSample 1 CSM4589A2F	Uncategorize BioSample 1 CSM4589A3F	Uncategorized Sample BioSample 1 CSM4589A4RW3_JF_D11.RAW (F035247)
END OF FILE	TRUE	TRUE	DNA replicati	MCM6_YEAS	113 kDa	TRUE		unknown	12	17	20	27
2	TRUE	TRUE	Cell division	CDC54_YEAS	105 kDa	TRUE		unknown	22	10	10	10
3	TRUE	TRUE	DNA replicati	MCM2_YEAS	99 kDa	TRUE		unknown	6	12	14	9
4	TRUE	TRUE	Uncharacteri	YJ16_YEAST	68 kDa			unknown	10	12	12	7
5	TRUE	TRUE	Minichromos	MCM5_YEAS	86 kDa	TRUE		unknown	5	5	4	5
6	TRUE	TRUE	DNA replicati	MCM3_YEAS	108 kDa			unknown	1	2	5	8
7	TRUE	FALSE	Cell division	A6ZLE4_YEA	95 kDa			unknown	1	2	0	4
END OF FILE												

SUMMARY

		Bottom band	2	3	Top band 4
113 kDa	M6	12	17	20	27
105 kDa	M4	22	10	10	10
99 kDa	M2	6	12	14	9
68 kDa	Cdt1	10	12	12	7
86 kDa	M5	5	5	4	5
108 kDa	M3	1	2	5	8
95 kDa	M7	1	2	0	4

Supplementary Table 1. Summary of Mass spectrometry data from protein cross-linking experiments.

Table S2 X-ray data collection and refinement statistics.

	Cdt1(1-438) Crystal form 1	Cdt1(1-438) Crystal form 2	Cdt1(495-604)
**Data collection:**			
Wavelength (Å)	0.97934	0.97856	0.97246
Space group	$P2_12_12_1$	$P2_12_12_1$	$P2_12_12_1$
Unit cell parameters			
a, b, c (Å)	82.9, 122.5, 148.4	47.5, 89.35, 121.9	43.9, 85.9, 89.0
α, β, γ (°)	90, 90, 90	90, 90, 90	90, 90, 90
Number of crystals used	1	1	1
Resolution (Å)	74-2.70 (2.85-2.70) [a]	42-2.15 (2.26-2.15)	45-1.80 (1.85-1.80)
Number of reflections			
measured	474,554	155,597	576,345
unique	42,303	26,255	31,963
Completeness (%)	100 (100)	98.7 (95.4)	99.6 (98.9)
Multiplicity	11.2 (11.6)	5.8 (5.8)	18.0 (18.4)
$\langle I/\sigma(I) \rangle$	15.8 (2.2)	13.1 (2.0)	20.2 (1.9)
R_{merge}	0.12 (1.40)	0.098 (1.06)	0.11 (1.91)
R_{pim} (all I^+ and I^-)	0.038 (0.409)	0.043 (0.464)	0.03 (0.46)
**SAD phasing statistics:**			
No. of Se sites refined	23		17
FOM (autosol)	0.28		0.34
Model-map CC (autosol)	0.49		0.82
**Refinement statistics:**			
Resolution range (Å)	74-2.70 (2.80-2.70)	42-2.15 (2.23-2.15)	45-1.80 (1.86-1.80)
Number of reflections			
work set	42,217	28,345	31,905
free	4,056	1,433	3,047
R_{work}/R_{free}	0.199/0.231	0.203/0.249	0.185/0.210
Number of atoms			
total	9,216	3,294	2,155
protein	9,071	3,092	1,826
ligands	53	26	10
solvent	92	176	319
R.m.s. deviations from ideal			
bond lengths (Å)	0.003	0.002	0.013
bond angles (°)	0.77	0.61	1.25
Average B-factor (Å^2)	81.4	56.1	31.6
Ramachandran plot (%) [b]			
favored	97	98	97
disallowed	0	0	0

[a] Values in parentheses correspond to the highest resolution bin.
[b] Analyzed using MolProbity (http://molprobity.biochem.duke.edu/).

Supplementary Table 2. Summary of X-ray crystallography data

Strain	Genotype	Reference
yLD70	*MATa ade2-1 ura3-1 his3-11 trp1-1::TRP1bar1 leu2-3::LEU2pep4 can1-100*	This study
yJF8	*MATa ade2-1 ura3-1 his3-11 trp1-1::TRP1bar1 leu2-3::LEU2pep4 can1-100 MCM2::MCM2-GFP(HIS3)*	This study
yJF9	*MATa ade2-1 ura3-1 his3-11 trp1-1::TRP1bar1 leu2-3::LEU2pep4 can1-100 MCM4::MCM4-GFP(HIS3)*	This study
yJF10	*MATa ade2-1 ura3-1 his3-11 trp1-1::TRP1bar1 leu2-3::LEU2pep4 can1-100 MCM6::MCM6-GFP(HIS3)*	This study
yJF28	*MATa ade2-1 ura3-1 his3-11 trp1-1::TRP1bar1 leu2-3::LEU2pep4 can1-100 MCM7::MCM7-GFP(HIS3)*	This study
yJF29	*MATa ade2-1 ura3-1 his3-11 trp1-1::TRP1bar1 leu2-3::LEU2pep4 can1-100 MCM3::MCM3-GFP(HIS3)*	This study
yJF12	*MATa ade2-1 ura3-1 his3-11 trp1-1::TRP1bar1 leu2-3::LEU2pep4 can1-100 MCM2::MCM2-GFP(HIS3) MCM2-GFP::MCM2-GFP-3XFLAG(KANMX)*	This study
yJF13.1	*MATa ade2-1 ura3-1 his3-11 trp1-1::TRP1bar1 leu2-3::LEU2pep4 can1-100 MCM4::MCM4-GFP(HIS3) MCM4-GFP::MCM4-GFP 3XFLAG(KANMX)*	This study
yJF14	*MATa ade2-1 ura3-1 his3-11 trp1-1::TRP1bar1 leu2-3::LEU2pep4 can1-100 MCM6::MCM6-GFP(HIS3) MCM6-GFP::MCM6-GFP-3XFLAG(KANMX)*	This study
yJF30	*MATa ade2-1 ura3-1 his3-11 trp1-1::TRP1bar1 leu2-3::LEU2pep4 can1-100 MCM7::MCM7-GFP(HIS3) MCM7-GFP::MCM7-GFP-3XFLAG(KANMX)*	This study
yJF31	*MATa ade2-1 ura3-1 his3-11 trp1-1::TRP1bar1 leu2-3::LEU2pep4 can1-100 MCM3::MCM3-GFP(HIS3) MCM3-GFP::MCM3-GFP-3XFLAG(KANMX)*	This study
yJF59	W303 1a pep4::KanMx4 bar1::HphNT1 his3-11::[HIS3pJF2::Cdt1-3xFLAG::NATNT2] trp1-1::TRP1pJF3leu2-3::LEU2pJF4 ura3-1::URA3pJF5	Frigola et al., 2013
yJF39	*W303-1a pep4::KanMx4 bar1::Hph-NT1 his3-11::HIS3pJF2 trp1-1::TRP1pJF3*	Frigola et al., 2013

	leu2-3::LEU2pJF4 ura3-1::URA3pJF6 CDT1::CDT1-TAP^{TCP}(HIS3)	
SD-ORC	*W303-1a pep4::KanMx4 bar1:: Hph-NT1 his3-11::HIS3pJF17 trp1-1::TRP1pJF18 ura3-1::URA3pJF19*	Frigola et al., 2013
SD-ORC(Δ6)	*W303-1a pep4::KanMx4 bar1:: Hph-NT1 his3-11::HIS3pJF17 trp1-1::TRP1pJF18 ura3-1::URA3pJF19 ORC6:: ORC6-3xFlag (Nat-NT2)*	Frigola et al., 2013

Supplementary Table 3. Yeast strains used in this study.

Plasmid	Cloning Vector	Insert	Reference
pMAL-M6N	pMAL-C2P	*MCM6 1-1461bp*	This study
pMAL-M6C1	pMAL-C2P	*MCM6 1462-3051bp*	This study
pMAL-M6C2	pMAL-C2P	*MCM6 2367-3051bp*	This study
pET22b-M6ΔC	pET22b	*MCM6 1-2616bp*	This study
pETS-CNM	Champion pET SUMO	*CDT1 1-1314bp*	This study
pETS-CMC	Champion pET SUMO	*CDT1 813-1812bp*	This study
pETS-CM1	Champion pET SUMO	*CDT1 813-1314bp*	This study
pETS-CM2	Champion pET SUMO	*CDT1 813-1488bp*	This study
pETS-CC1	Champion pET SUMO	*CDT1 1485-1812bp*	This study
pETS-CC3	Champion pET SUMO	*CDT1 1308-1812bp*	This study

Supplementary Table 4. Plasmids used in this study.

Primer	Sequence	Target	Direction
JF25	CGC GGA TCC ATG TCA TCC CCT TTT CCA	*MCM6*	FOR
JF26	CCG AGG GTC GAC TTA ATT AGC AGC CAT	*MCM6*	REV
JF27	GGC GGA TCC ATG TTA CAA GCT AAT AAT	*MCM6*	FOR
JF28	CTG GAC GTC GAC TTA GCT GGA ATC CTG	*MCM6*	REV
JF29	GCG GGA TCC ATG AGT AGA TCG AGT TAT	*MCM6*	FOR
JF32	GGTATGAGTGGCACAGCTAATAG	*CDT1*	FOR
JF33	TCAATTAGAGCTGGACCTTC	*CDT1*	REV
JF34	GGTATGAGTAAAGGAGAGGGTAC	*CDT1*	FOR
JF35	TCAATCTTGTTGTTTTGATTTGTG	*CDT1*	REV
JF36	TCAATTAGAGCTGGACCTTC	*CDT1*	REV
JF37	TCATGCGGCCGAACGTCTCTC	*CDT1*	REV
JF38	GGTATGGCCGCATTGCTTTCTC	*CDT1*	FOR
JF39	TCAATCTTGTTGTTTTGATTTGTG	*CDT1*	REV
JF40	GGTATGGTACCCGCTTTCAAAATA	*CDT1*	FOR
JF41	GTGTTCGCCGCCAACTCCGCAGGTCTTTCG TACCTTGGGTCACCGTACGCTGCAGGTCGA	*MCM2*	FOR
JF42	GAATTTTTTATCTTCATATCCAGATATTCG TAGGAATAACAAAGTTTTAATCGATGAATT CGAGCTCG	*MCM2*	REV
JF43	TGTCGGACAGAAATAACTTAATGGTTGCT GACGATAAAGTTTGGAGAGTCCGTACGCT GCAGGTCGAC	*MCM3*	FOR
JF44	AAAGCCAAGAATGGAAGTCTTTTAGTAAA CATTCCTGTGACATCAATCGATGAATTCG AGCTCG	*MCM3*	REV
JF45	TTGTCCTTGGCGAGGGTGTAAGGAGATCA GTTCGCCTGAATAACCGTGTCCGTACGCT GCAGGTCGAC	*MCM4*	FOR
JF46	TAGTATTTATTAATTGTTACGCAGGGAATG ATTGTAGTAGACAGCATCAATCGATGAAT TCGAGCTCG	*MCM4*	REV
JF47	TTCATCCAAACTGTGAGGTTTTGGATCAAT TGGAACCACAGGATTCCAGCCGTACGCT GCAGGTCGAC	*MCM6*	FOR
JF48	GAACGATACTTGAAACGAAATATGAAAAT CCGCAAGAGTGCACTGAATTAATCGATGA ATTCGAGCTCG	*MCM6*	REV
JF49	TTCCGCTAATGTGAGCGCCCAAGATTCTG ATATCGATCTACAAGACGCTCGTACGCTG CAGGTCGAC	*MCM7*	FOR
JF50	AAAGAATAAAGAATGAAGGCCCTGTTGCT TTTTTTTTTAGAACTTTCAATCGATGAATTC GAGCTCG	*MCM7*	REV
JF51	AGTGTTCGCCGCCAACTCCGCAGGTCTTT CGCAATTTATACCTTGGGTCACCGTACGC	*MCM2*	FOR

	TGCAGGTCGAC		
JF52	CACTATAGGGCGAATTGGAGCTCCACCG CGGTGGCGGCCGCGCTTTATCAATCGAT GAATTCGAGCTCG	*pYM44*	REV
JF53	TTGAAGATCATGTCGGACAGAAATAACTT AATGGTTGCTGACGATAAAGTTTGGAGAG TCCGTACGCTGCAGGTCGAC	*MCM3*	FOR
JF54	GGTCATTGTCCTTGGCGAGGGTGTAAGG AGATCAGTTCGCCTGAATAACCGTGTCCG TACGCTGCAGGTCGAC	*MCM4*	FOR
JF55	CCAAACTGTGAGGTTTTGGATCAATTGGA ACCACAGGATTCCAGCCGTACGCTGCAG GTCGAC	*MCM6*	FOR
JF56	CGTCCTGCTCCCGGGGCTTCAAGCGTCTTG TAGATCGATATCAGAATC	*MCM7*	FOR
NC7	GGAAATGCATATGTCATCCCCTTTTCCAG CTGACAC	*MCM6*	FOR
NC8	CCGCTCGAGTCATGACCCAGTCCCATCAT CATTGTC	*MCM6*	REV

Supplementary Table 5. Oligonucleotides used in this study.

Supplementary Methods

Yeast Strains and Vectors

Mcm6 fragments.

Mcm6 fragments were amplified by PCR using JF25-26, 27-28 and 29-28 primer pairs. These PCR products were cloned into pMAL-C2P to yield pMAL-Mcm6N, pMAL-Mcm6C1 and pMAL-Mcm6C2 between BamHI and Sal I. pMAL-C2P was derived from pMAL-C2 (NEB) by introducing a PreScission protease site before the EcoRI site in the polylinker region.

Mcm6ΔC.

Mcm6ΔC was amplified by PCR using NC7-8 primer pairs. This PCR product was cloned into pET22b (Invitrogen), between NdeI and XhoI, yielding in pET22b-Mcm6ΔC.

Cdt1 fragments.

Cdt1 fragments were amplified using JF32-33, 34-35, 34-36, 34-37, 38-39 and 40-39. This PCR products were cloned into champion pET SUMO (Invitrogen) leading to pETS-CNM, CMC, CM1, CM2, CC1 and CC3 respectively.

MCM GFP,FLAG-Cdt1 complexes.

Using yLD70 as a background strain, Mcm2, 3, 4, 6 and 7 were GFP tagged using PCR products obtained from JF41-42, 43-44, 45-46, 47-48, 49-50 primer pairs and pYMcm44 vector as a template, yielding into yJF8, 29, 9, 10 and 28, respectively. An additional 3xFLAG tag was added using JF51-52, 53-52, 54-52, 55-52 and 56-52 primer pairs, yielding to yJF12, 31, 13.1, 14 and 30, respectively.

Protein Purification

Mcm6 fragments.

All expression plasmids were transformed into BL21 DE3 Codon+ RIL cells (Stratagene). 1.5 L of cells were grown at 37°C to a density of OD_{600}=0.5-0.8. Cells were chilled on ice, and then IPTG was added to 1 mM. Induction was carried out O/N at 18ºC. Cells were harvested, washed once with ice-cold 25 mM Hepes-KOH pH7.6,1M sorbitol, once with buffer C (50 mM Tris-HCl pH 7.5, 0.05% NP-40, 10% glycerol), 1 M NaCl and then the pellet was resuspended in 20 ml of buffer C plus 1M NaCl, 2 mM ß-mercapethanol, protease inhibitors (Roche). 25 µl of lysozyme (50 mg/ml) were added and the suspension incubated for 20 minutes at 4ºC. Cells were kept on ice and sonicated 3 x 30 sec at 15 microns using a sonicator Soniprep 150 (Sanyo). Lysate was centrifuged for 1 hour at 45,000 rpm using a Ti45 rotor. The soluble phase was collected and incubated with 1 ml packed amylose bead volume (NEB) at 4ºC for 1 hour. Beads were washed with ten BVs of buffer C plus 0.3 M NaCl, 2 mM ß-mercaptoethanol. Elution was performed with buffer C plus 0.3 M NaCl, 2 mM ß-mercaptoethanol, 10 mM maltose. Peak fractions were pooled and 0.5 ml were subjected to fractionation over a Superdex 200 10/300 GL column (GE Healthcare) pre-equilibrated in buffer C plus 0.3 M NaCl, 2 mM ß-mercaptoethanol. Peak fractions were diluted with buffer C plus 2 mM ß-mercaptoethanol, pooled and concentrated on 0.15 ml MonoQ™ PC1.6/5 column (GE Healthcare), using an elution gradient of 0.15-0.5 M NaCl over 15 CVs. Peak fractions containing MBP-Mcm6 fragments were kept.

Mcm6ΔC protein.

Cells were grown and expressed as described above. Cells were harvested, washed once with ice-cold 25 mM Hepes-KOH pH 7.6/1 M sorbitol, once with buffer D (50 mM Tris-HCl pH 7.5, 0.05% NP-40, 10% glycerol,1mM EDTA and 1mM DTT) plus 1 M NaCl, and the pellet was resuspended in 40 ml of buffer D plus1M NaCl, protease inhibitors (Roche). Cells were sonicated and the lysate centrifuged for 1 hour at 45000 rpm using a Ti45 rotor. The soluble fraction from centrifugation was treated

with 0.3 g/ml ammonium sulphate, and stirred for 20 minutes at 4ºC. The mixture was centrifuged at 17,000 rpm for 20 minutes. The pellet was resuspended in 40 ml of buffer D containing 0.25 g/ml of ammonium sulphate and centrifuged as before. This was repeated with 0.20 mg/ml ammonium sulphate. The pellet was resuspended in 30 ml of buffer D and dialyzed against buffer D for one hour at 4ºC. After checking that the conductivity of the sample was below that of buffer D plus 0.1 M NaCl, the sample was applied to a 5 ml FF Q column (GE Healthcare), pre-equilibrated in buffer D plus 0.1 M NaCl. Elution was done using a gradient of 0.1 M to 0.5 M NaCl over 10 CVs. Peak fractions were pooled and subjected to fractionation over a Superdex 200 10/300 GL column (GE Healthcare) pre-equilibrated with buffer D plus 0.3 M NaCl. Peak fractions were diluted with buffer D and subjected to 0.15 ml MonoQ™ PC1.6/5 column (GE Healthcare), using an elution gradient of 0.15-0.5 M NaCl over 15 CVs. Peak fractions containing Mcm6ΔC were kept.

Cdt1 fragments.

All expression plasmids were transformed into E. coli Rosetta2(DE3) cells (Novagen). 3 L of cells were grown at 30°C to a density of OD_{600}=0.7-0.9. Protein expression was induced with 1mM IPTG for 4 hours. Cells were harvested by centrifugation. Cells were resuspended in buffer E (25mM Tris-HCl, pH 7.4) plus 0.5M NaCl,1mM PMSF and disrupted using sonication. Sample was centrifuged for 30 minutes at 42,000rpm (Ti45 rotor). Supernatant was collected and 20mM imidazole was added. His_6-tagged proteins were captured on His trap FF crude 5 ml column (Amersham) and eluted with buffer E plus 0.5M NaCl, 200mM imidazole. His_6-SUMO tag was removed by overnight digestion with His_6-tagged catalytic core domain of *S. cerevisiae* Ulp1, at 4ºC with buffer E plus 0.3M NaCl. Non cleaved and Ulp1 proteins were removed using His trap FF crude 5 ml column (Amersham) and the flow through was diluted with buffer E and fractionated using 5ml Hi Trap FF Q column (GE healthcare) in the case of Cdt1 NM fragment, while the other Cdt1

fragments were subjected to Hi Trap FF SP column (GE healthcare) instead. Proteins were eluted with a linear 0.1-0.5M NaCl gradient in 25mM Tris-HCl, pH 7.4. Peak fractions were pooled and loaded into HiLoad 16/60 Superdex 200 column (GE healthcare) pre equilibrated with buffer E plus 0.1M NaCl, 2mM DTT and 10% glycerol was added to the samples and snap frozen in liquid nitrogen.

Cdt1-FLAG.

yJF59 strain overexpresses MCM-Cdt1 FLAG. During the fractionation of this complex in a HiLoad 16/60 Superdex 200 column (GE healthcare), later fractions eluted from the column containing Cdt1-FLAG as a monomer were pooled and stored.

Mcm2, Mcm7 and Mcm3.

To purify these proteins, we followed the protocol described by Davey et al., 2003 (ref 2). The starting cultures were 3 L in volume.

Mcm5, Mcm6, Mcm4 and Cdt1.

We followed the protocol described by Frigola et al., 2013 (ref 3). The starting cultures were 3 L in volume.

SD-ORC, yJF38, yJF39, yJF59 and Cdc6.

We followed the protocol described by Frigola et al., 2013 (ref 3).

MCM GFP,FLAG -Cdt1 complexes.

We followed the protocol described by Remus et al., 2009 (ref 1).

Supplementary References

1. Remus, D. et al. Concerted loading of Mcm2-7 double hexamers around DNA during DNA replication origin licensing. *Cell* **139**, 719-30 (2009).
2. Davey, M.J., Indiani, C. & O'Donnell, M. Reconstitution of the Mcm2-7p heterohexamer, subunit arrangement, and ATP site architecture. *J Biol Chem* **278**, 4491-9 (2003).
3. Frigola, J., Remus, D., Mehanna, A. & Diffley, J.F.X. ATPase-dependent quality control of DNA replication origin licensing. *Nature* **495**, 339-43 (2013).
